# Gene Suppression of Transketolase-Like Protein 1 (TKTL1) Sensitizes Glioma Cells to Hypoxia and Ionizing Radiation

**DOI:** 10.3390/ijms19082168

**Published:** 2018-07-25

**Authors:** Sonja Heller, Gabriele D. Maurer, Christina Wanka, Ute Hofmann, Anna-Luisa Luger, Ines Bruns, Joachim P. Steinbach, Johannes Rieger

**Affiliations:** 1 Dr. Senckenberg Institute of Neurooncology and University Cancer Center (UCT), University Hospital Frankfurt, Goethe University, 60590 Frankfurt am Main, Germany; sonja.heller@kgu.de (S.H.); gabriele.maurer@kgu.de (G.D.M.); christina.wanka@gmx.de (C.W.); anna-luisa.luger@kgu.de (A.-L.L.); i.hartel@web.de (I.B.); j.rieger@uni-tuebingen.de (J.R.); 2 German Cancer Research Center (DKFZ) Heidelberg, German Cancer Consortium (DKTK), Partner Site Frankfurt/Mainz, 60590 Frankfurt am Main, Germany; 3 Dr. Margarete Fischer-Bosch Institute of Clinical Pharmacology, Stuttgart, Eberhard Karls University, 72074 Tuebingen, Germany; ute.hofmann@ikp-stuttgart.de; 4 Interdisciplinary Division of Neuro-Oncology, Hertie Institute for Clinical Brain Research, University Hospital Tuebingen, Eberhard Karls University, 72076 Tuebingen, Germany

**Keywords:** transketolase-like protein 1, pentose phosphate pathway, glioma, hypoxia, radiation, metabolism

## Abstract

In several tumor entities, transketolase-like protein 1 (TKTL1) has been suggested to promote the nonoxidative part of the pentose phosphate pathway (PPP) and thereby to contribute to a malignant phenotype. However, its role in glioma biology has only been sparsely documented. In the present in vitro study using LNT-229 glioma cells, we analyzed the impact of *TKTL1* gene suppression on basic metabolic parameters and on survival following oxygen restriction and ionizing radiation. *TKTL1* was induced by hypoxia and by hypoxia-inducible factor-1α (HIF-1α). Knockdown of *TKTL1* via shRNA increased the cells’ demand for glucose, decreased flux through the PPP and promoted cell death under hypoxic conditions. Following irradiation, suppression of *TKTL1* expression resulted in elevated levels of reactive oxygen species (ROS) and reduced clonogenic survival. In summary, our results indicate a role of TKTL1 in the adaptation of tumor cells to oxygen deprivation and in the acquisition of radioresistance. Further studies are necessary to examine whether strategies that antagonize TKTL1 function will be able to restore the sensitivity of glioma cells towards irradiation and antiangiogenic therapies in the more complex in vivo environment.

## 1. Introduction

Due to their high proliferation rate, malignant cells exhibit increased energy turnover; this characteristic is the basis for therapeutic strategies such as conventional chemo- and radiotherapy and some imaging techniques such as positron emission tomography. Tumorigenesis and tumor progression are associated with metabolic alterations and this re-programming of metabolic pathways has been found to profoundly impact cellular behavior, tumor macro- and microenvironment. Transketolase-like protein 1 (TKTL1) catalyzes the conversion of sedoheptulose 7-phosphate and d-glyceraldehyde 3-phosphate to d-ribose 5-phosphate and d-xylulose 5-phosphate [1], thereby contributing to a more active pentose phosphate pathway (PPP). As the majority of the cell’s ribose 5-phosphate [2], used for nucleic acid synthesis, and NADPH, required for biosynthetic reactions as well as for neutralizing reactive oxygen species (ROS), are provided by the PPP, a crucial role of TKTL1 in malignant cell biology has been proposed. In addition to its transketolase function, protective effects towards oxidative stress and apoptosis have been shown to exist independently of its enzymatic activity [3]. Consistent with these attributes, an increased expression of TKTL1 on the mRNA and/or protein level has been reported in several tumor entities, including glioblastoma [4] and colon cancer [5]. In colon carcinoma cell lines, *TKTL1* expression was induced by hypoxia [6]. We have previously shown that suppressing *TKTL1* expression in glioma cells increases ROS under hypoxic conditions and antagonizes the protection against hypoxia-induced cell death conferred by TP53-induced glycolysis and apoptosis regulator (TIGAR) [7]. However, other studies [8] and publicly available databases such as the Human Protein Atlas [9] and the R2 database (Genomics Analysis and Visualization Platform, http://r2.amc.nl) do not show abundant TKTL1 protein levels or *TKTL1* expression in gliomas. Such inconsistent findings might be due to either different methodological approaches or to context-specific regulation of transcription or translation in different subpopulations and environmental conditions. In particular, oxygen availability in tumors is known to fluctuate temporally and spatially [10], and hypoxia is closely linked to malignant progression and resistance to therapeutic approaches in a variety of solid tumors [11,12]. In our present study, we therefore analyzed the effects of *TKTL1* gene silencing with special regard to hypoxic conditions.

## 2. Results

### 2.1. Hypoxia and HIF-1α Enhance TKTL1 Expression

**Figure 1:**
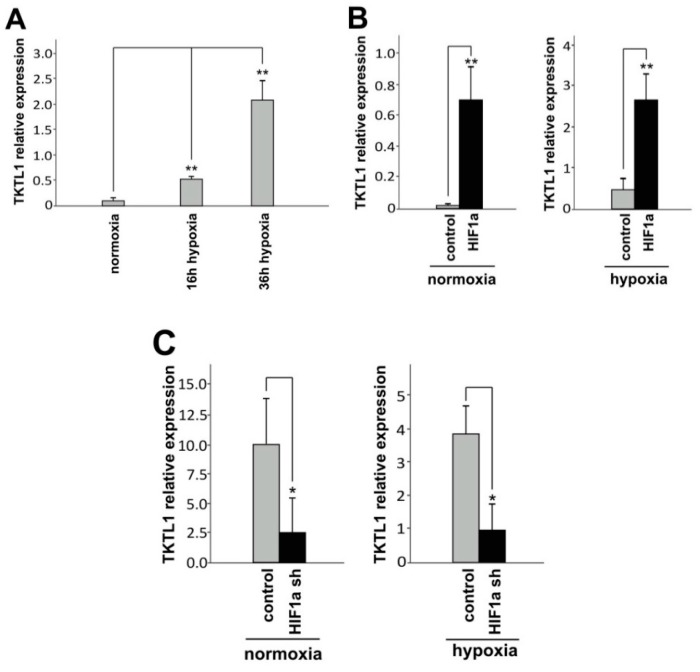
Hypoxia and HIF-1α upregulate *TKTL1* expression. (**A**) LNT-229 cells were grown at normoxia for 36 h and at hypoxia for 16 h and 36 h, respectively, and *TKTL1* expression was analyzed by RT-qPCR (mean + SD, ** *p* < 0.01); (**B**) LNT-229 cells were transiently transfected with pcDNA3-HIF-1α or pcDNA3 control and 24 h later subjected to different oxygen concentrations. Another 24 h later, *TKTL1* was assessed by RT-qPCR (mean + SD, ** *p* < 0.01); (**C**) similarly, LNT-229 cells stably expressing shRNA targeting *HIF-1α* or its *Drosophila* homolog *Sima* (control) were grown in normoxia and hypoxia and 24 h later analyzed for *TKTL1* mRNA levels (mean + SD, * *p* < 0.05).

In LNT-229 glioma cells used for our experiments, *TKTL1* was upregulated under hypoxic conditions (Figure 1A). As hypoxia-inducible factor-1α (HIF-1α) is known to be a key regulator of the cellular response to hypoxia, we modified the availability of *HIF-1α* and then analyzed *TKTL1* expression. Overexpression of *HIF-1α* increased *TKTL1* (Figure 1B) whereas *HIF-1α* knockdown reduced *TKTL1* (Figure 1C).

### 2.2. TKTL1 Gene Silencing Reduces Levels of Sedoheptulose 7-Phosphate

**Figure 2:**
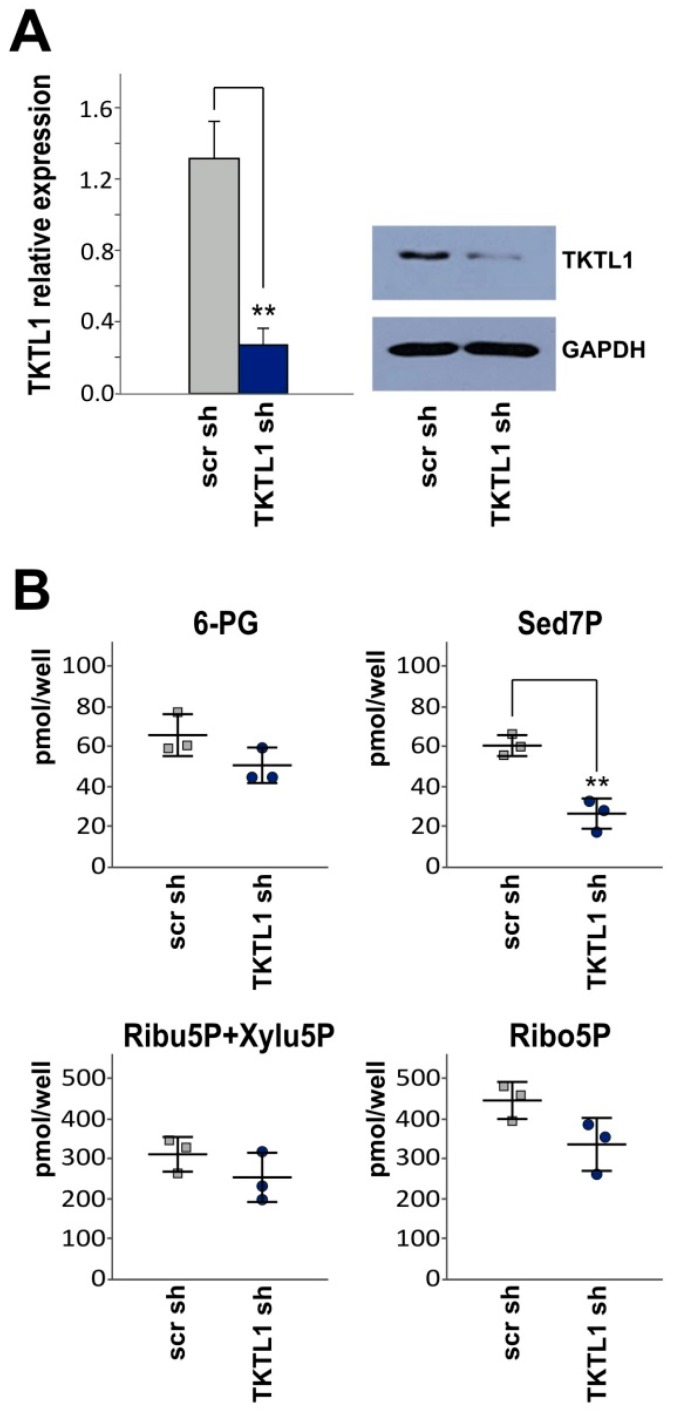
shRNA-mediated suppression of *TKTL1* expression reduces intracellular content of sedoheptulose 7-phosphate. (**A**) shRNA-mediated *TKTL1* suppression was verified by RT-qPCR (delta CT value, control, 10.08 and delta CT value, LNT-229-shTKTL1, 12.38) and western blot analysis; (**B**) LNT-229-shTKTL1 and control (scr) cells were analyzed for intracellular PPP metabolites 6-phosphogluconate (6-PG), sedoheptulose 7-phosphate (Sed7P), ribulose 5-phosphate (Ribu5P), xylulose 5-phosphate (Xylu5P) and ribose 5-phosphate (Ribo5P), ** *p* < 0.01.

In order to assess the impact of TKTL1 on basic metabolic characteristics, we generated LNT-229 cells stably expressing shRNA targeting *TKTL1* and a scrambled shRNA sequence, respectively, and verified the knockdown by RT-qPCR and western blot analysis (Figure 2A). Metabolomic profiling revealed a significant decrease in sedoheptulose 7-phosphate following *TKTL1* knockdown (Figure 2B). Suppression of *TKTL1* thus attenuated the amount of this PPP intermediate, indicating a flux shift away from PPP and e.g., towards glycolysis. However, levels of 6-phosphogluconate, ribulose 5-phosphate, xylulose 5-phosphate and ribose 5-phosphate did not change significantly.

### 2.3. TKTL1 Knockdown Raises Glucose Consumption and Lactate Production in Hypoxia

**Figure 3:**
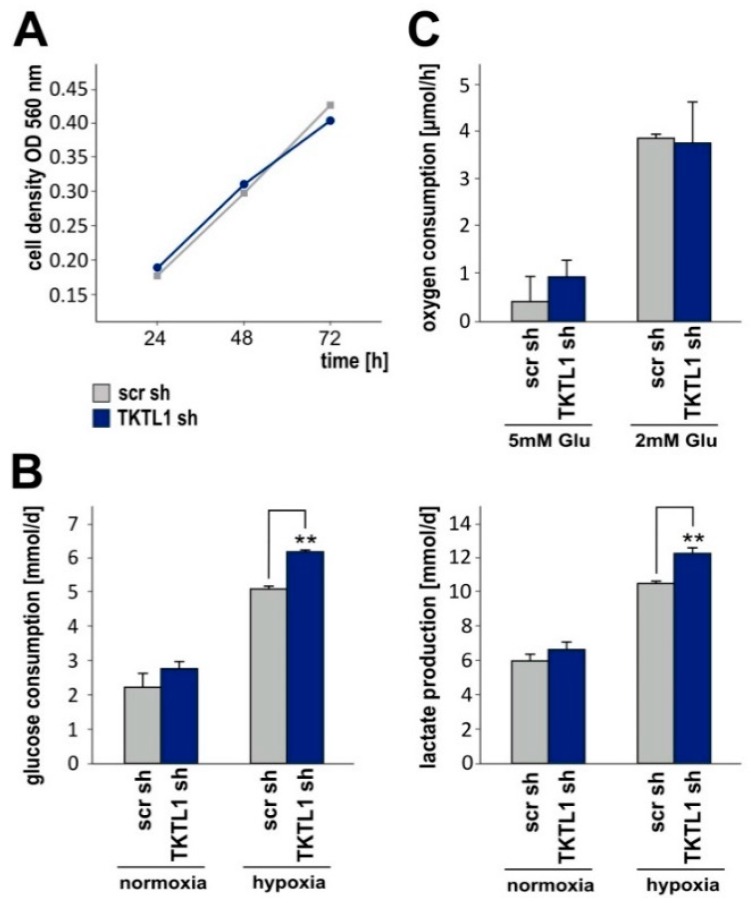
*TKTL1* knockdown enhances glucose consumption and lactate production under hypoxic conditions. (**A**) LNT-229-shTKTL1 and control (scr) cells were cultured in normoxia. Cell density was assessed by crystal violet staining after 24 h, 48 h and 72 h; (**B**) cells were seeded in medium supplemented with 10% FCS and 25 mM glucose and 24 h later exposed to serum-free medium containing 6.5 mM glucose and normoxia or hypoxia. Another 24 h later, glucose and lactate in supernatants were measured (mean + SD, ** *p* < 0.01); (**C**) oxygen consumption was calculated by incubating the cells in serum-free medium containing 2 mM or 5 mM glucose for 8 h and using the OxoDish^R^ system (mean + SD).

Stable suppression of *TKTL1* did not alter cell density as assessed by crystal violet staining over a period of up to 72 h (Figure 3A). Accordingly, potential differences between LNT-229-shTKTL1 and control cells in subsequent analyses of basic metabolic parameters should not be due to different growth rates. Moreover, we performed analyses over a short period of time to minimize more subtle effects of proliferation. In normoxia, glucose consumption and lactate production did not differ between cells expressing normal and reduced levels of *TKTL1*. By contrast, *TKTL1* gene silencing increased both glucose consumption and lactate production under hypoxic conditions (Figure 3B). However, oxygen consumption rates did not vary significantly between LNT-229-shTKTL1 and control cells (Figure 3C), nor did concentrations of fumarate, malate and citrate, intermediates of the tricarboxylic acid cycle (Figure S1).

### 2.4. TKTL1 Knockdown Enhances Intracellular ROS Levels and Augments Cell Death during Oxygen Restriction

**Figure 4:**
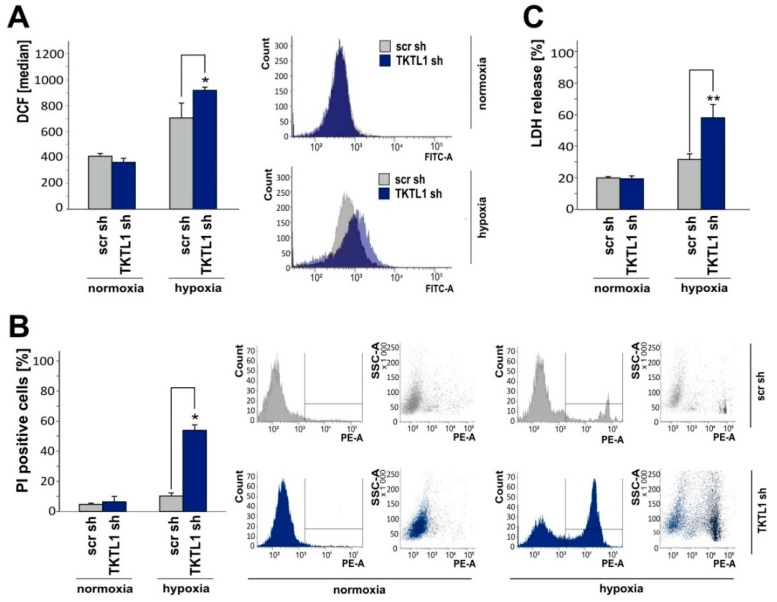
In hypoxia, *TKTL1* gene silencing increases reactive oxygen species (ROS) levels and cell death. (**A**) LNT-229-shTKTL1 and control (scr) cells were cultured in serum-free medium supplemented with 5 mM glucose under normoxic or hypoxic conditions for 24 h. Thereafter, intracellular ROS were evaluated using H_2_DCFDA and flow cytometry (median fluorescence intensity, mean + SD, * *p* < 0.05). Using medium containing 2 mM glucose, cell death was assessed by PI staining after a 36 h incubation ((**B**), mean percentage of PI-positive cells + SD, * *p* < 0.05) and by quantification of LDH release after a 72 h incubation ((**C**), mean + SD, ** *p* < 0.01).

ROS are formed as byproducts of aerobic metabolism and involved in the regulation of cell proliferation, differentiation, apoptosis, inflammation and aging [13]. Excessive ROS levels, as induced by some chemotherapeutic agents and ionizing radiation, result in cell death when exceeding the reduction capacity of cancerous or healthy tissue. Therefore, maintaining a reduction-oxidation (redox) balance is crucial for tumor cells to sustain pro-survival signaling pathways and to prevent cell death [14]. Using the ROS-sensitive dye dichlorodihydrofluorescein diacetate (H_2_DCFDA) and flow cytometry, we observed an increase in ROS following *TKTL1* gene silencing in hypoxia. No impact of *TKTL1* on intracellular ROS was detectable under normoxic conditions (Figure 4A). Similarly, knockdown of *TKTL1* promoted cell death in hypoxia but not in normoxia, as assessed by propidium iodide (PI) staining (Figure 4B) and lactate dehydrogenase (LDH) release (Figure 4C).

### 2.5. TKTL1 Gene Silencing Sensitizes Cells to Ionizing Radiation

**Figure 5:**
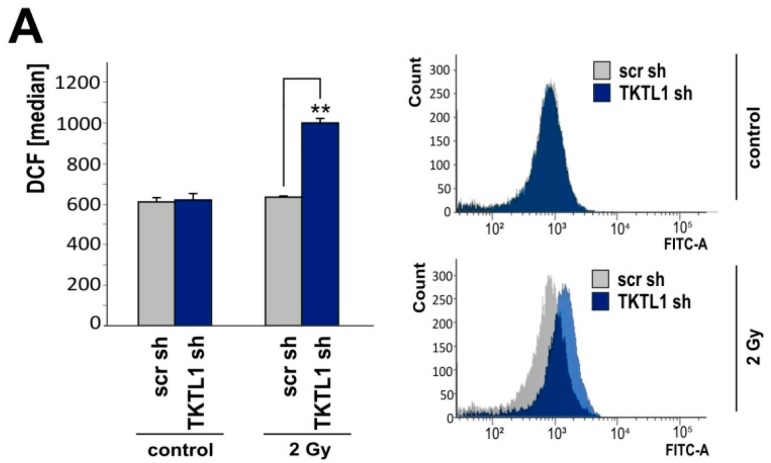

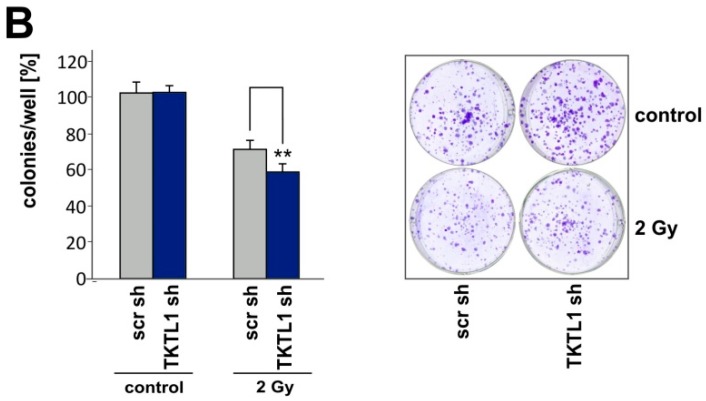
*TKTL1* knockdown elevates ROS levels and impairs clonogenic survival subsequent to irradiation. (**A**) LNT-229-shTKTL1 and control (scr) cells were seeded in medium supplemented with 10% FCS and 25 mM glucose, 24 h later irradiated with a photon dose of 2 Gy and another 6 h later analyzed for ROS levels (median fluorescence intensity, mean + SD, ** *p* < 0.01); (**B**) cells were plated at very low density (500 cells in six-well plates), 24 h later irradiated and thereafter monitored for clonogenic survival (mean number of colonies, displayed as percentages of non-irradiated controls, + SD, ** *p* < 0.01).

Radiotherapy induces the formation of ROS and is a key component of glioblastoma treatment. Cells exposed to 2 Gy irradiation were examined for ROS levels and monitored for clonogenic survival. LNT-229-shTKTL1 cells exhibited more ROS (Figure 5A) and displayed less clonogenic survival than control cells (Figure 5B).

### 2.6. TKTL1 Knockdown in HCT-116 Cells Produces Similar Effects

**Figure 6:**
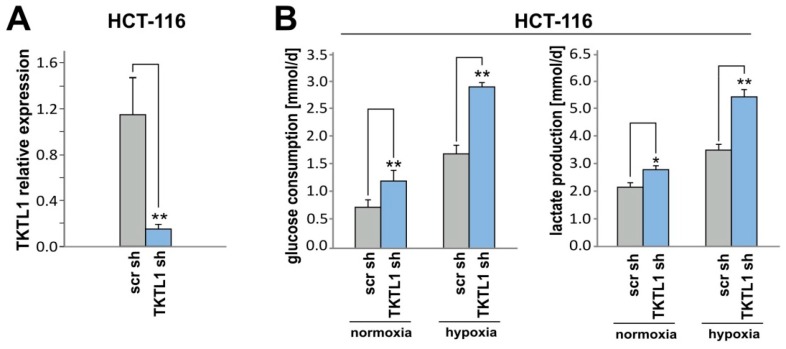

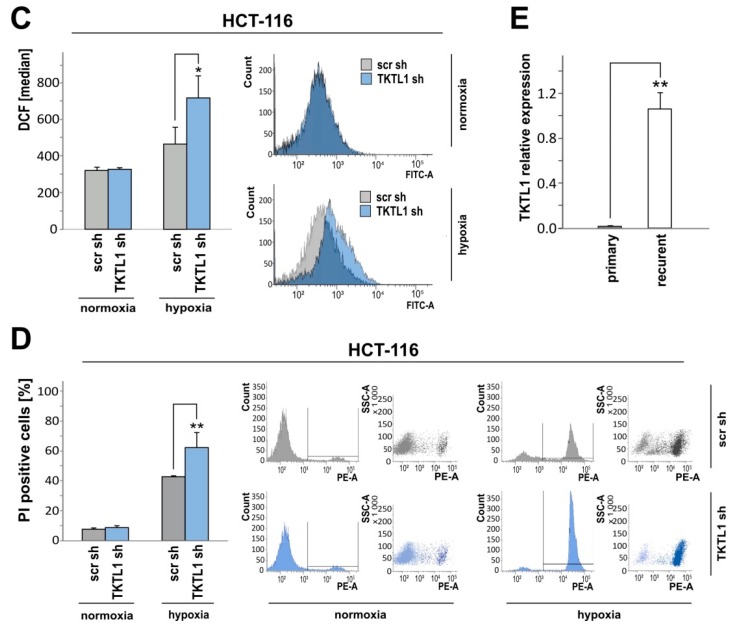
Following *TKTL1* knockdown, HCT-116 cells consume more glucose, generate more lactate, and under hypoxic conditions, accumulate more ROS and display less clonogenic survival. (**A**) In HCT-116 cells, shRNA-mediated *TKTL1* suppression was verified by RT-qPCR. HCT-116-shTKTL1 and control (scr) cells were exposed to serum-free medium containing 6.5 mM glucose and normoxia or hypoxia. 24 h later, glucose and lactate in supernatants were measured ((**B**), mean + SD, * *p* < 0.05, ** *p* < 0.01), and intracellular ROS levels were assessed ((**C**), median fluorescence intensity, mean + SD, * *p* < 0.05). After a 30 h incubation in medium supplemented with 2 mM glucose, cell viability was evaluated by PI staining ((**D**), mean percentage of PI-positive cells + SD, ** *p* < 0.01); (**E**) *TKTL1* expression in cultured cells from a patient’s primary and recurrent glioblastoma was examined by RT-qPCR (mean + SD of three independent experiments using cells originating from the same surgical specimens, ** *p* < 0.01).

To verify the data shown above, we repeated key experiments using another shRNA sequence and received consistent results (data not shown). Additionally, we analyzed HCT-116 colon carcinoma cells and again applied two different shRNA sequences targeting *TKTL1* (Figure 6A and data not shown). HCT-116-shTKTL1 cells consumed more glucose and produced more lactate than the corresponding control cells (Figure 6B). In contrast to LNT-229-shTKTL1 cells, they did so both in normoxia and in hypoxia. Similar to our observations in LNT-229 cells, *TKTL1* gene silencing in HCT-116 cells increased ROS levels (Figure 6C) and cell death (Figure 6D) specifically under hypoxic conditions. ROS analysis and transient knockdown of *TKTL1* in T98G cells confirmed this finding in another glioma cell line (Figure S2). Hypoxia-induced changes are known to contribute to tumor recurrence [15,16,17,18,19]. We therefore examined *TKTL1* expression in cultured cells derived from a patient’s primary and recurrent glioblastoma. At relapse, an impressive increase in *TKTL1* mRNA levels was detected in comparison to the initial diagnosis (Figure 6E).

## 3. Discussion

**Figure 7:**
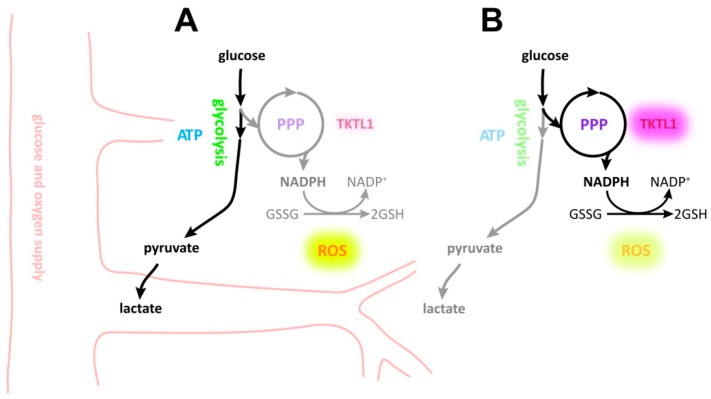
Outline of hypoxia-mediated effects on TKTL1 and subsequent metabolic bias. (**A**) In normoxia, *TKTL1* expression does not impact ROS production and survival; (**B**) in the absence of adequate oxygen supply, *TKTL1* is upregulated, accentuating the throughput of the PPP and resulting in lower ROS levels and finally less cell death.

Since the first description of TKTL1 by Coy et al. in 1996 [1,20], its role in health and disease remains to be defined. TKTL1 has been proposed to accelerate the nonoxidative PPP [21] and to contribute to the malignant phenotype in a variety of neoplasms. Very little data is available concerning gliomas. An overexpression of TKTL1 in glioma, as well as a correlation with tumor grade has been reported [4]. We found that (1) LNT-229 glioma cells expressed TKTL1, (2) *TKTL1* expression was upregulated in hypoxia and depended on the presence of *HIF-1α*, (3) *TKTL1* suppression was accompanied by a downregulation of PPP intermediate sedoheptulose 7-phosphate, by an elevated glucose turnover and higher lactate levels indicating accelerated glycolysis and by an increase in intracellular ROS under hypoxic conditions, and (4) *TKTL1* knockdown facilitated hypoxia-induced cell death and lowered clonogenic survival following irradiation. Figure 7 illustrates the changes effected by TKTL1 depending on oxygen conditions.

An induction of *TKTL1* by hypoxia has been described in T84 and Caco-2 cells, derived from a lung metastasis of human colon carcinoma and from a colon carcinoma, respectively [6]. Applying siRNA targeting *TKTL1* in nasopharyngeal carcinoma cell lines CNE2 and HONE1, Dong and Wang reported a drop in cell viability and in levels of NADPH and ribose 5-phosphate [22]. Combining *TKTL1* suppression and cisplatin, they delineated additive cytotoxic effects. Further, *TKTL1* knockdown inhibited human hepatoma HepG2 cell proliferation [23]. The impact of *TKTL1* inhibition on cell viability apparently depends on cell type-specific characteristics. Administering temozolomide, another DNA-damaging agent, and assessing cell density and clonogenic survival, we did not detect any difference between LNT-229-shTKTL1 and control cells (data not shown). Our findings do not basically contradict those of Dong and Wang as in our system *TKTL1* knockdown per se was not associated with a loss of viability, and we noticed neither synergistic nor antagonistic effects of *TKTL1* suppression on the activity of temozolomide. In contrast, oxythiamine, an antimetabolite and transketolase inhibitor, exhibited cytotoxicity in our LNT-229 cell line (data not shown). Xu et al. also used human HCT-116 colon carcinoma cell line and a similar shRNA-based technique, and found decreases in cell growth, glucose consumption and lactate production [24]. As they selected stable single-cell clones while we employed stable transfection pools, the conflicting results of their experiment and ours might be due, at least in part, to clonal effects. In colon cancer, increasing expression of TKTL1 has been associated with local progression at the primary tumor site (T1–2 versus T3–4) whereas patients presenting with distant metastasis (M1) had (primary) tumors expressing less TKTL1 than those of M0-patients [25]. Anaerobic metabolism becomes more important the more the primary tumor expands and cells are moved away from continuous oxygen supply. Our data indicate that TKTL1 makes cells less susceptible to hypoxia-induced cell death, for example by reducing their glucose requirements and increasing ROS detoxification. Our observation that *TKTL1* was upregulated in a case of recurrent glioblastoma in comparison to the primary tumor is compatible with a role of TKTL1 in the process of tumor evolution in vivo, with tumor hypoxia, which is known to increase at recurrence [26], as the primary selective pressure. However, larger numbers of samples need to be investigated before definite conclusions can be drawn from this finding. Taken together, under anaerobic circumstances, malignant cells may benefit from *TKTL1* expression and the presence or absence of such conditions could account for the inconsistent reports on the significance of TKTL1 in tumor biology.

In tumors, hypoxia arises from an imbalance of rapidly proliferating cells and blood supply. Furthermore, some types of treatment, e.g., bevacizumab, a humanized monoclonal antibody against vascular endothelial growth factor (VEGF)-A, induce hypoxia [27]. Hypoxia causes stabilization of HIFs [28] and increases [29] or decreases [30] the expression of genes involved in the PPP. An activated PPP, in turn, may promote malignant transformation, protect from apoptosis and favor migration [31]. The current literature on both TKTL1 and PPP does not yield uniform results and observations vary in a cell type and context-specific manner. Our present study therefore expands the scarce data on the role of TKTL1 in malignant glioma. We did not address the way metabolism is modified by TKTL1 and whether its mechanism of action is predominantly dependent or independent of its enzymatic activity. However, we provide evidence that TKTL1 renders cells more resistant to radiation therapy and to hypoxic conditions. Strategies targeting TKTL1 expression therefore could restore or boost the therapeutic effect of irradiation and antiangiogenic agents and are worth further investigation.

## 4. Materials and Methods

### 4.1. Reagents, Cell Lines and Culture Conditions

Unless otherwise specified, reagents were purchased from Sigma-Aldrich (St. Louis, MO, USA) or Qiagen (Hilden, Germany). Antibodies used were anti-glyceraldehyde-3-phosphate dehydrogenase (GAPDH, MAB374, Chemicon, Nürnberg, Germany) and anti-TKTL1 (GeneTex, Irvine, CA, USA), shRNA sequences were TKTL1, TRCN0000055696 (GACAATCTTGTGGCAATCTTT) and TRCN0000415911 (TTCATCCCTAGTTCGGAAATT), and scrambled control 5′-GATCCCCACTACCGTTGT-TATAGGTCTTCAAGAGAGACCTATAACAACG-GTAGTTTTTTGGAAA-3′, siRNA sequences were TKTL1, antisense UAAAUAACCAUAGUUUCUGGU, sense ACCAGAAACUAUGGUUAUUUA [24], and antisense UUAUUCACGAAGGAAACACUU, sense AAGUGUUUCCUUCGUGAAUAA [24], and AllStars negative control siRNA (Qiagen), vectors introduced were pcDNA3 control (Invitrogen, Carlsbad, CA, USA) and pcDNA3-HIF-1α (Addgene, Cambridge, MA, USA). T98G human malignant glioma cells, obtained from the ATCC (Manassas, VA, USA), LNT-229 human malignant glioma cells, kindly provided by N. de Tribolet, LNT-229 cells stably expressing an shRNA targeting *HIF-1α* and its control (*Sima*), kindly provided by T. Acker [32], and HCT-116 colon carcinoma cells, acquired from the ATCC, were expanded in Dulbecco’s modified Eagle’s Medium (4500 mg/L glucose), supplemented with 10% fetal calf serum (FCS; PAA, Pasching, Austria), 2 mM glutamine, 100 IU/mL penicillin, 100 μg/mL streptomycin and if required (LNT-229-shHIF-1α and control cells) 10 µg/mL blasticidin, at 37 °C and 5% CO_2_. For some experiments, serum- and glucose-free medium was supplemented with glucose to concentrations of 2 or 5 mM. After transfection using Attractene (Qiagen), cells stably expressing shRNA constructs targeting *TKTL1* and scrambled control sequences, respectively, were selected by puromycin resistance (5 µg/mL). Hypoxia was generated by using Gas Pak pouches for anaerobic culture (Becton-Dickinson, Heidelberg, Germany). For irradiation experiments, cells were exposed to single doses of 2 Gy photons using a linear accelerator (SL75/5, Elekta, Crawley, UK) with 6 MeV/100 cm focus-surface distance and a dose rate of 4 Gy/min. 0 Gy-controls were kept in parallel at ambient temperature in the accelerator control room.

### 4.2. SDS-PAGE and Immunoblotting

Lysis buffer comprised 50 mM Tris-HCl, 120 mM NaCl, 5 mM EDTA, 0.5% Nonidet P-40, 2 μg/mL aprotinin, 10 μg/mL leupeptin, 100 μg/mL phenylmethylsulfonyl fluoride, 50 mM NaF, 200 μM NaVO5 and phosphatase inhibitor cocktails I and II. Following estimation of protein content using a Bradford assay (Bio-Rad, Hercules, CA, USA), 20 μg of total protein was separated under reducing conditions by sodium dodecyl sulfate polyacrylamide gel electrophoresis (SDS-PAGE) and electroblotted on nitrocellulose (Amersham, Braunschweig, Germany). Membranes were blocked in Tris-buffered saline containing 5% skim milk and 0.1% Tween-20 and incubated with the appropriate primary (dilution 1:1000) and secondary (dilution 1:3000) antibodies. Immune complexes were detected by enhanced chemiluminescence (Pierce, Rockford, IL, USA).

### 4.3. Real-Time Quantitative PCR (RT-qPCR)

Total RNA was extracted using TRIzol™ (Invitrogen) and the RNeasy™ system (Qiagen), cDNA was synthesized using 1.5 μg of RNA and SuperScript VILO™ (Invitrogen). RNA extraction from a patient’s primary and recurrent glioblastoma after surgical resection was done with the patient’s written informed consent and with the approval of the institutional review board (UCT Frankfurt) and the ethics committee (University Hospital Frankfurt, project number SNO_SIN_02-08, ethics committee vote 04/09_SNO_01/08, 17 March 2009). For real-time PCR, ABsolute™ Blue qPCR SYBR-Green Fluorescein Mix (Thermo Fisher Scientific, Waltham, MA, USA) and an iQ5 real-time PCR detection system (Bio-Rad, Munich, Germany) were employed. Gene expression data were normalized to the internal control succinate dehydrogenase complex, subunit A, flavoprotein variant (SDHA) using the ddCT method and the iQ5 software (version 2.1, Bio-Rad, Munich, Germany). Primer sequences: SDHA forward 5′-TGGGAACAAGAGGGCATCTG-3′ and reverse 5′-CCACCACTGCATCAAATTCATG-3′, TKTL1 forward 5′-TAACACCATGACGCCTACTGC-3′ and reverse 5′-CATCCTAACAAGCTTTCGCTG-3′.

### 4.4. Measurement of Glucose Uptake, Lactate Production and Oxygen Consumption

Cell-free supernatants were checked for glucose and lactate concentrations in a Hitachi 917 analyzer (Roche Diagnostics, Mannheim, Germany). Oxygen consumption was determined applying OxoDish^R^ 24-well plates (PreSens, Regensburg, Germany) and carefully overlaying cells with sterile paraffin oil.

### 4.5. Quantification of Intracellular Metabolites

Cells were seeded, allowed to adhere overnight and then incubated for 8 h in serum-free medium containing 2 mM glucose. Further analysis was performed as described previously [33,34,35].

### 4.6. ROS Analysis

Intracellular ROS were determined by flow cytometric analysis of H_2_DCFDA (Invitrogen) [36].

### 4.7. Growth and Viability Assays

Cell density was evaluated by crystal violet staining, resolubilizing the dye in 0.1 M sodium citrate and measuring the absorbance at 560 nm (Multiskan™ EX; Thermo Fisher Scientific, Langenselbold, Germany).

Cell death was quantified by propidium iodide (PI) staining of adherent and non-adherent cells (1 µg/mL) and flow cytometry (BD Canto II, Heidelberg, Germany). Cytotoxicity was assayed by measuring the amount of released lactate dehydrogenase (LDH) with the Cytotoxicity Detection Kit (LDH, Roche). Both techniques, PI staining and LDH release, were based on the loss of membrane integrity [37]. PI is a nucleic acid intercalating dye that cannot pass through intact cell membranes. It is therefore commonly used for identifying late apoptotic and necrotic cells within a population at a pre-defined time. When the cell membrane is damaged, LDH, a soluble yet stable intracellular enzyme, is released into the culture medium. LDH reduces NAD+ to NADH/H+ via the oxidation of lactate to pyruvate. Then, diaphorase transfers H/H+ from NADH/H+ to the tetrazolium salt INT which is reduced to the red colored formazan. The amount of formazan dye formed is quantified spectroscopically at 490 nm and proportional to the number of lysed cells.

Clonogenic survival was studied by seeding 500 cells in six-well plates, observing them for 7 days, staining them with crystal violet und counting colonies of more than 50 cells.

### 4.8. Statistics

Experiments were repeated at least three times with similar results. Results are depicted as mean + standard deviation (SD) and differences were considered significant if *p* < 0.05 using the two-tailed Student’s *t*-test.

## Supplementary Materials

The supplementary materials are available online at http://www.mdpi.com/1422-0067/19/8/2168/s1.

## References

[1] Coy J.F., Dressler D., Wilde J., Schubert P. (2005). Mutations in the transketolase-like gene TKTL1: Clinical implications for neurodegenerative diseases, diabetes and cancer. Clin. Lab..

[2] Boros L.G., Puigjaner J., Cascante M., Lee W.N., Brandes J.L., Bassilian S., Yusuf F.I., Williams R.D., Muscarella P., Melvin W.S. (1997). Oxythiamine and dehydroepiandrosterone inhibit the nonoxidative synthesis of ribose and tumor cell proliferation. Cancer Res..

[3] Diaz-Moralli S., Aguilar E., Marin S., Coy J.F., Dewerchin M., Antoniewicz M.R., Meca-Cortés O., Notebaert L., Ghesquière B., Eelen G. (2016). A key role for transketolase-like 1 in tumor metabolic reprogramming. Oncotarget.

[4] Völker H.-U., Hagemann C., Coy J., Wittig R., Sommer S., Stojic J., Haubitz I., Vince G.H., Kämmerer U., Monoranu C.-M. (2008). Expression of transketolase-like 1 and activation of Akt in grade IV glioblastomas compared with grades II and III astrocytic gliomas. Am. J. Clin. Pathol..

[5] Langbein S., Zerilli M., Zur Hausen A., Staiger W., Rensch-Boschert K., Lukan N., Popa J., Ternullo M.P., Steidler A., Weiss C. (2006). Expression of transketolase TKTL1 predicts colon and urothelial cancer patient survival: Warburg effect reinterpreted. Br. J. Cancer.

[6] Bentz S., Cee A., Endlicher E., Wojtal K.A., Naami A., Pesch T., Lang S., Schubert P., Fried M., Weber A. (2013). Hypoxia induces the expression of transketolase-like 1 in human colorectal cancer. Digestion.

[7] Wanka C., Steinbach J.P., Rieger J. (2012). Tp53-induced glycolysis and apoptosis regulator (TIGAR) protects glioma cells from starvation-induced cell death by up-regulating respiration and improving cellular redox homeostasis. J. Biol. Chem..

[8] Kämmerer U., Gires O., Pfetzer N., Wiegering A., Klement R.J., Otto C. (2015). TKTL1 expression in human malign and benign cell lines. BMC Cancer.

[9] Uhlen M., Zhang C., Lee S., Sjöstedt E., Fagerberg L., Bidkhori G., Benfeitas R., Arif M., Liu Z., Edfors F. (2017). A pathology atlas of the human cancer transcriptome. Science.

[10] Horsman M.R., Vaupel P. (2016). Pathophysiological Basis for the Formation of the Tumor Microenvironment. Front. Oncol..

[11] Muz B., de la Puente P., Azab F., Azab A.K. (2015). The role of hypoxia in cancer progression, angiogenesis, metastasis, and resistance to therapy. Hypoxia.

[12] Eales K.L., Hollinshead K.E.R., Tennant D.A. (2016). Hypoxia and metabolic adaptation of cancer cells. Oncogenesis.

[13] Schieber M., Chandel N.S. (2014). ROS function in redox signaling and oxidative stress. Curr. Biol..

[14] Moloney J.N., Cotter T.G. (2018). ROS signalling in the biology of cancer. Semin. Cell Dev. Biol..

[15] Yang L., Lin C., Wang L., Guo H., Wang X. (2012). Hypoxia and hypoxia-inducible factors in glioblastoma multiforme progression and therapeutic implications. Exp. Cell Res..

[16] Thiepold A.-L., Luger S., Wagner M., Filmann N., Ronellenfitsch M.W., Harter P.N., Braczynski A.K., Dützmann S., Hattingen E., Steinbach J.P. (2015). Perioperative cerebral ischemia promote infiltrative recurrence in glioblastoma. Oncotarget.

[17] Bette S., Barz M., Huber T., Straube C., Schmidt-Graf F., Combs S.E., Delbridge C., Gerhardt J., Zimmer C., Meyer B. (2018). Retrospective Analysis of Radiological Recurrence Patterns in Glioblastoma, Their Prognostic Value And Association to Postoperative Infarct Volume. Sci. Rep..

[18] Stadlbauer A., Mouridsen K., Doerfler A., Bo Hansen M., Oberndorfer S., Zimmermann M., Buchfelder M., Heinz G., Roessler K. (2018). Recurrence of glioblastoma is associated with elevated microvascular transit time heterogeneity and increased hypoxia. J. Cereb. Blood Flow Metab..

[19] McIntyre A., Harris A.L. (2015). Metabolic and hypoxic adaptation to anti-angiogenic therapy: A target for induced essentiality. EMBO Mol. Med..

[20] Coy J.F., Dübel S., Kioschis P., Thomas K., Micklem G., Delius H., Poustka A. (1996). Molecular cloning of tissue-specific transcripts of a transketolase-related gene: Implications for the evolution of new vertebrate genes. Genomics.

[21] Patra K.C., Hay N. (2014). The pentose phosphate pathway and cancer. Trends Biochem. Sci..

[22] Dong Y., Wang M. (2017). Knockdown of TKTL1 additively complements cisplatin-induced cytotoxicity in nasopharyngeal carcinoma cells by regulating the levels of NADPH and ribose-5-phosphate. Biomed. Pharmacother..

[23] Zhang S., Yang J.-H., Guo C.-K., Cai P.-C. (2007). Gene silencing of TKTL1 by RNAi inhibits cell proliferation in human hepatoma cells. Cancer Lett..

[24] Xu X., Zur Hausen A., Coy J.F., Löchelt M. (2009). Transketolase-like protein 1 (TKTL1) is required for rapid cell growth and full viability of human tumor cells. Int. J. Cancer.

[25] Diaz-Moralli S., Tarrado-Castellarnau M., Alenda C., Castells A., Cascante M. (2011). Transketolase-like 1 expression is modulated during colorectal cancer progression and metastasis formation. PLoS ONE.

[26] Scholz A., Harter P.N., Cremer S., Yalcin B.H., Gurnik S., Yamaji M., Di Tacchio M., Sommer K., Baumgarten P., Bähr O. (2016). Endothelial cell-derived angiopoietin-2 is a therapeutic target in treatment-naive and bevacizumab-resistant glioblastoma. EMBO Mol. Med..

[27] Hattingen E., Jurcoane A., Bähr O., Rieger J., Magerkurth J., Anti S., Steinbach J.P., Pilatus U. (2011). Bevacizumab impairs oxidative energy metabolism and shows antitumoral effects in recurrent glioblastomas: A 31P/1H MRSI and quantitative magnetic resonance imaging study. Neuro-Oncology.

[28] Wigerup C., Påhlman S., Bexell D. (2016). Therapeutic targeting of hypoxia and hypoxia-inducible factors in cancer. Pharmacol. Ther..

[29] Gao L., Mejías R., Echevarría M., López-Barneo J. (2004). Induction of the glucose-6-phosphate dehydrogenase gene expression by chronic hypoxia in PC12 cells. FEBS Lett..

[30] Kathagen-Buhmann A., Schulte A., Weller J., Holz M., Herold-Mende C., Glass R., Lamszus K. (2016). Glycolysis and the pentose phosphate pathway are differentially associated with the dichotomous regulation of glioblastoma cell migration versus proliferation. Neuro-Oncology.

[31] Riganti C., Gazzano E., Polimeni M., Aldieri E., Ghigo D. (2012). The pentose phosphate pathway: An antioxidant defense and a crossroad in tumor cell fate. Free Radic. Biol. Med..

[32] Henze A.-T., Riedel J., Diem T., Wenner J., Flamme I., Pouyseggur J., Plate K.H., Acker T. (2010). Prolyl hydroxylases 2 and 3 act in gliomas as protective negative feedback regulators of hypoxia-inducible factors. Cancer Res..

[33] Hofmann U., Maier K., Niebel A., Vacun G., Reuss M., Mauch K. (2008). Identification of metabolic fluxes in hepatic cells from transient 13C-labeling experiments: Part I. Experimental observations. Biotechnol. Bioeng..

[34] Maier K., Hofmann U., Reuss M., Mauch K. (2010). Dynamics and control of the central carbon metabolism in hepatoma cells. BMC Syst. Biol..

[35] Thiepold A.-L., Lorenz N.I., Foltyn M., Engel A.L., Divé I., Urban H., Heller S., Bruns I., Hofmann U., Dröse S. (2017). Mammalian target of rapamycin complex 1 activation sensitizes human glioma cells to hypoxia-induced cell death. Brain.

[36] Wanka C., Brucker D.P., Bähr O., Ronellenfitsch M., Weller M., Steinbach J.P., Rieger J. (2012). Synthesis of cytochrome C oxidase 2: A p53-dependent metabolic regulator that promotes respiratory function and protects glioma and colon cancer cells from hypoxia-induced cell death. Oncogene.

[37] Vanden Berghe T., Grootjans S., Goossens V., Dondelinger Y., Krysko D.V., Takahashi N., Vandenabeele P. (2013). Determination of apoptotic and necrotic cell death in vitro and in vivo. Methods.

