# Supplementary material for: Gene Suppression of Transketolase-Like Protein 1 (TKTL1) Sensitizes Glioma Cells to Hypoxia and Ionizing Radiation
Source: Int J Mol Sci. 2018 Jul 25;19(8):2168. doi: 10.3390/ijms19082168 (PMC6121283; doi:10.3390/ijms19082168)
Supplement: Supplementary file 1 [file ijms-19-02168-s001.zip › ijms-326006-SI.pdf]

Gene Suppression of Transketolase-Like Protein 1 (TKTL1) Sensitizes Glioma Cells to Hypoxia and Ionizing Radiation

Sonja Heller, Gabriele D. Maurer, Christina Wanka, Ute Hofmann, Anna-Luisa Luger, Ines Bruns, Joachim P. Steinbach, Johannes Rieger

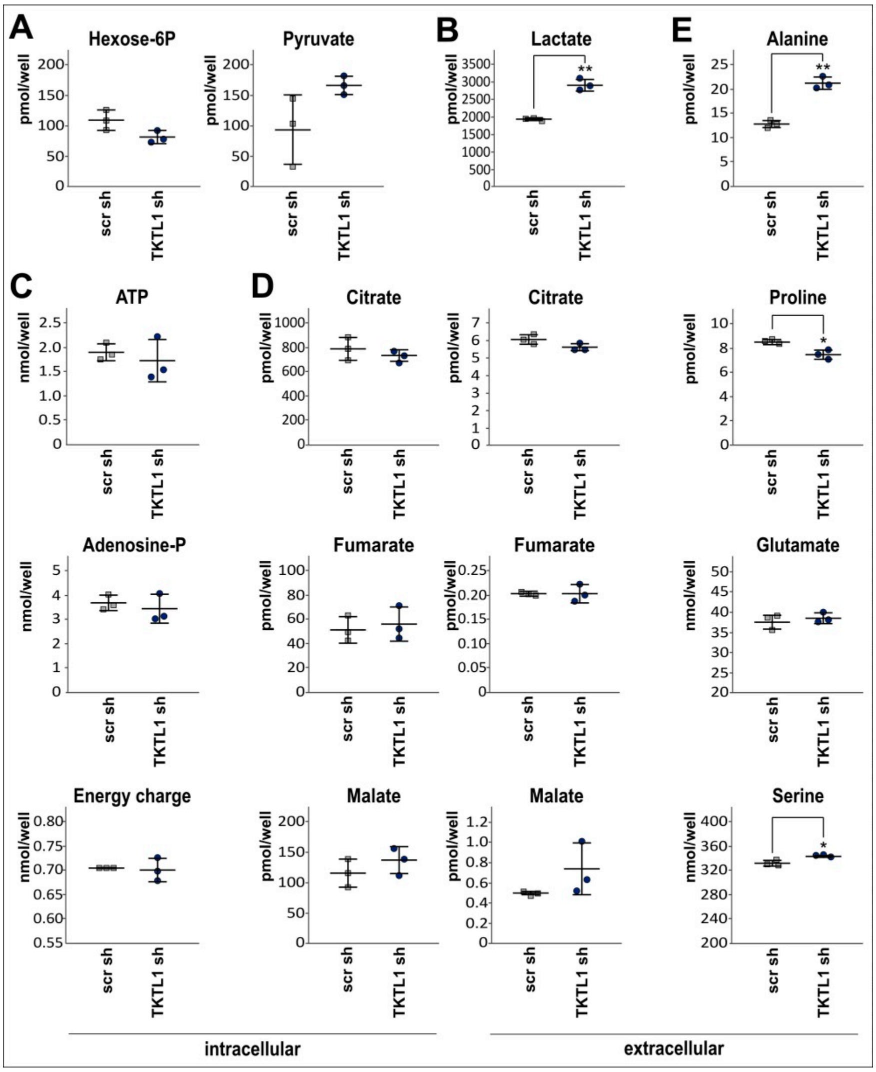

Figure S1. Analysis of selected metabolic parameters in LNT-229-shTKTL1 and control cells under normoxic conditions. (A) Intracellular concentrations of hexose-6-phosphate (Hexose-6P) and pyruvate did not differ significantly, whereas (B) extracellular lactate levels were found to be higher in LNT-229-shTKTL1 cells (**$p < 0.01$). (C) Quantities of adenosine triphosphate (ATP) and adenosine phosphate (Adenosine-P), as well as the calculated energy charge (ATP + 0.5*ADP) / (ATP + ADP + AMP) [1], were not different either. (D) Concentrations of fumarate, malate and citrate, intermediates of the tricarboxylic acid cycle, were measured both intra- and extracellularly. (E) Further, the extracellular content of amino acids alanine, proline, glutamate and serine was monitored.

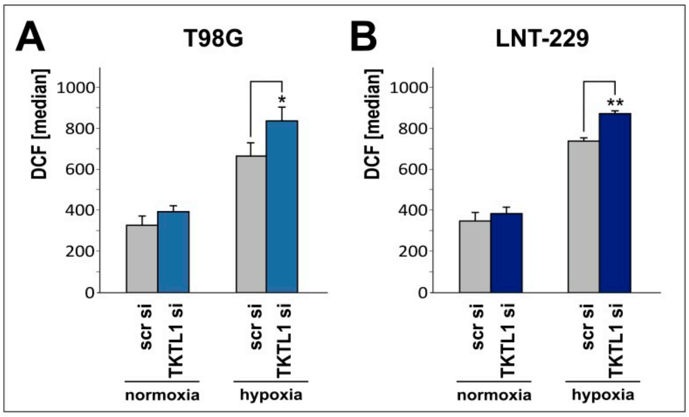

Figure S2. *TKTL1* gene suppression raises ROS levels under hypoxic conditions. T98G (A) and LNT-229 (B) cells were transiently transfected with 20 nM of either scrambled control siRNA or siRNA targeting *TKTL1*. 24 h later, cells were exposed to serum-free medium containing 5 mM glucose and to normoxic or hypoxic conditions. Another 20 h later, intracellular ROS generation was estimated using H_2DCFDA and flow cytometry (median fluorescence intensity, mean + SD, **p* < 0.05, ***p* < 0.01).

References

1. Atkinson, D.E.; Walton, G.M. Adenosine triphosphate conservation in metabolic regulation. Rat liver citrate cleavage enzyme. *J. Biol. Chem.* **1967**, *242*, 3239–3241.
